# PW01-021 – The phenotype of FMF due to deletion M694

**DOI:** 10.1186/1546-0096-11-S1-A74

**Published:** 2013-11-08

**Authors:** HJ Lachmann, DM Rowczenio, JA Gilbertson, JD Gillmore, AD Wechalekar, T Lane, PN Hawkins

**Affiliations:** 1 University College London Medical School, London, UK; 2 National Amyloidosis Centre, University College London Medical School, London, UK

## Introduction

Autosomal dominant FMF due to deletion of residue 694 was first described in 3 British families in 2000. It has subsequently been reported in other patients of Northern European ancestry but the phenotype has not been fully characterised.

## Objectives

To describe the presenting symptoms, complications and treatment responses of familial Mediterranean fever due to deletion M694.

## Methods

We sought patients with a genetic finding of deletion M694 from our database and reviewed their case records.

## Results

A total of 19 patients (11 M:8 F) had been found to carry the Del M694 variant. Clinical details were available on 16 patients who had been assessed at our centre. 13 were of white British ancestry, the other 3 were of Irish ancestry. 2 patients gave no relevant family history, 1 was adopted and unaware of any family details, 10 patients (from 5 kindreds) gave a history of similar symptoms in at least 1 relative and the final patient reported that his mother died of renal failure of unknown cause raising the possibility of AA amyloidosis although without suggestive symptoms. 13 patients had symptoms: median age at onset 18 years (range 6-48), median age at diagnosis 48.1 years, median attack duration 2.5 days with a median of 1 attack per month, all described fever with peritonitic abdominal pain, pleuritic symptoms occurred in 4 cases, and erysipelas like erythema rash in 2. 3 patients had an appendectomy and 2 cholecystectomy prior to diagnosis. 5 of 7 symptomatic women reported attacks with menstruation and 3 had partial remissions with oral contraception. 3 patients had presented with AA amyloidosis of unrecognised aetiology of whom only 1 was truly asymptomatic. 2 children detected as part of family screening also denied any symptoms. 11 patients are on colchicine with good clinical and inflammatory responses, median age at starting treatment was 50 years, 2 felt that oral contraceptives provided adequate symptom relief, 1 declined treatment.

## Conclusion

Familial Mediterranean fever associated with deletion M694 is an autosomal dominant condition in Northern European Caucasians with variable penetrance. Disease onset appears slightly later but symptoms and colchicine responsiveness are very similar to classical autosomal recessive FMF. The high rate of AA amyloidosis (19%) may reflect the very delayed diagnosis and late initiation of colchicine treatment.

## Disclosure of interest

None declared

